# Supplementary material for: Effects of Short-Chain Fatty Acids on Human Oral Epithelial Cells and the Potential Impact on Periodontal Disease: A Systematic Review of In Vitro Studies
Source: Int J Mol Sci. 2020 Jul 11;21(14):4895. doi: 10.3390/ijms21144895 (PMC7402343; doi:10.3390/ijms21144895)
Supplement: Supplementary file 1 [file ijms-21-04895-s001.zip › Table S1.docx]

**Table S1** **-** Articles excluded and the reasons for exclusion (n = 25).

| Reference | Author | Reasons for Exclusion |
| --- | --- | --- |
|  | Chang et al. (2002) | 2 |
|  | Correa et al. (2015) | 1 |
|  | Correa et al. (2017) | 2 |
|  | Cueno and Ochiai (2016) | 1 |
|  | Eftimiadi et al. (1990) | 2 |
|  | Eftimiadi et al. (1991) | 2 |
|  | Hagi-Pavli et al. (2005) | 4 |
|  | Koseki et al. (1996) | 3 |
|  | Kurita-Ochiai et al. (1998) | 2 |
|  | Levine (1985) | 2 |
|  | Nebe et al. (2001) | 2 |
|  | Noguchi et al. (2000) | 4 |
|  | Pöllänen et al. (1997) | 2 |
|  | Robichaux et al. (2003) | 3 |
|  | Seto et al. (2008) | 2 |
|  | Shah et al. (1992) | 2 |
|  | Singer and Buckner (1981) | 2 |
|  | Tonetti et al. (1987) | 2 |
|  | Touw et al. (1982) | 2 |
|  | Uematsu et al. (2003) | 3 |
|  | Vinolo et al. (2009) | 2 |
|  | Wyss (2007) | 3 |
|  | Yin and Chung (2011) | 3 |
|  | Yoneda et al. (2013) | 3 |
|  | Yoshida et al (2015) | 3 |

Legend: 1 - Reviews, letters, posters, conference abstracts, case reports, animal studies, clinical studies, personal opinions; 2 - Studies in which cell lines used were not epithelial cells or were epithelial cells not derived from human oral tissues; 3 - Studies that focused on bacterial effects and not on short-chain fatty acids; 4 - Studies in which short-chain fatty acids were not applied as an intervention to oral epithelial cells; 5 - Studies with no control group; 6 - Studies in which periodontal disease was not an outcome.

**Table S1 - References**

1. Chang, Y.J.; Holtzman, M.J.; Chen, C.C. Interferon-gamma-induced epithelial ICAM-1 expression and monocyte adhesion. Involvement of protein kinase c-dependent c-Src tyrosine kinase activation pathway. *J. Biol. Chem*. **2002**, 277, 7118-26.

2. Correa, R.O.; Vieira, A.; Sernaglia, E.M.; Vinolo, M.A.R. Bacterial metabolites, short chain fatty acids, attenuate the immune response to aggregatibacter actinomycetemcomitans. *Inflamm. Res.* **2015**, 64(S2), S182-S183.

3. Corrêa, R.O.; Vieira, A.; Sernaglia, E.M.; Lancellotti, M.; Vieira, A.T.; Avila-Campos, M.J.; Rodrigues, H.G.; Vinolo, M.A.R. Bacterial short-chain fatty acid metabolites modulate the inflammatory response against infectious bacteria. *Cell. Microbiol*. **2017**, 19, e12720.

4. Cueno, M.E.; Ochiai, K. Re-discovering periodontal butyric acid: new insights on an old metabolite. *Microb. Pathog*. **2016**, 94, 48-53.

5. Eftimiadi, C.; Tonetti, M.; Massara, R.; Ferrarini, M.; Gandolfo, A.; Mangiante, P.E. Inhibition of the immune response due to the volatile fatty acids produced by anaerobic bacteria in the periodontal pocket. *Minerva Stomatol*. **1990**, 39, 357-60.

6. Eftimiadi, C.; Stashenko, P.; Tonetti, M.; Mangiante, P.E.; Massara, R.; Zupo, S.; Ferrarini, M. Divergent effect of the anaerobic bacteria by-product butyric acid on the immune response: suppression of T-lymphocyte proliferation and stimulation of interleukin-1 beta production. *Oral Microbiol. Immunol*. **1991**, 6, 17-23.

7. Hagi-Pavli, E.; Farthing, P.M.; Henshaw, F.N.; Kapas, S. Presentation of ICAM-1 protein at the cell surface of oral keratinocytes in the presence of adrenomedullin and corticotrophin. *Cell. Physiol. Biochem*. **2005**, 15, 167-74.

8. Koseki, T.; Ishikawa, I.; Boutsi, E.; He, T.; Benno, Y. Nutritional analysis and an enriched medium for fermentative treponemes isolated from subgingival plaque. *Oral Microbiol. Immunol*. **1996**, 11, 166-71.

9. Kurita-Ochiai, T.; Ochiai, K.; Fukushima, K. Volatile fatty acid, metabolic by-product of periodontopathic bacteria, induces apoptosis in WEHI 231 and RAJI B lymphoma cells and splenic B cells. *Infect. Immun*. **1998**, 66, 2587-94.

10. Levine, M. The role for butyrate and propionate in mediating HeLa-cells growth inhibition by human dental plaque fluid from adult periodontal disease. *Arch. Oral Biol*. **1985**, 30, 155-9.

11. Nebe, B.; Forster, C.; Pommerenke, H.; Fulda, G.; Behrend, D.; Bernewski, U.; Schmitz, K.P.; Rychly, J. Structural alterations of adhesion mediating components in cells cultured on poly-beta-hydroxy butyric acid. *Biomaterials*. **2001**, 22, 2425-34.

12. Noguchi, K.; Iwasaki, K.; Endo, H.; Kondo, H.; Shitashige, M.; Ishikawa, I. Prostaglandins E2 and I2 downregulate tumor necrosis factor alpha-induced intercellular adhesion molecule-1 expression in human oral gingival epithelial cells. *Oral Microbiol. Immunol*. **2000**, 15, 299-304.

13. Pöllänen, M.T.; Overman, D.O.; Salonen, J.I. Bacterial metabolites sodium butyrate and propionate inhibit epithelial cell growth in vitro. *J. Periodontal Res*. **1997**, 32, 326-34.

14. Robichaux, M.; Howell, M.; Boopathy, R. Methanogenic activity in human periodontal pocket. *Curr. Microbiol*. **2003**, 46, 53-8.

15. Seto, S.; Kurita-Ochiai, T.; Ochiai, T. Increased susceptibility to tumor necrosis factor-alpha in butyric acid-induced apoptosis is caused by downregulation of cFLIP expression in Jurkat T cells. *Microbiol. Immunol*. **2008**, 52, 188-96.

16. Shah, H.N.; Gharbia, S.E.; O'Toole, C.M. Assessment of the relative cytotoxicity of Porphyromonas gingivalis cells, products, and components on human epithelial cell lines. *J. Periodontol*. **1992**, 63, 44-51.

17. Singer, R.E.; Buckner, B.A. Butyrate and propionate: important components of toxic dental plaque extracts. *Infect. Immun*. **1981**, 32, 458-63.

18. Tonetti, M.; Eftimiadi, C.; Damiani, G.; Buffa, P.; Buffa, D.; Botta, G.A. Short chain fatty acids present in periodontal pockets may play a role in human periodontal diseases. *J. Periodontal Res*. **1987**, 22, 190-1.

19. Touw, J.J.; van Steenbergen, T.J.; De Graaff, J. Butyrate: A cytotoxin for Vero cells produced by Bacteroides gingivalis and Bacteroides asaccharolyticus. *Antonie Van Leeuwenhoek*. **1982**, 8, 315-25.

20. Uematsu, H.; Sato, N.; Hossain, M.Z.; Ikeda, T.; Hoshino, E. Degradation of arginine and other amino acids by butyrate-producing asaccharolytic anaerobic Gram-positive rods in periodontal pockets. *Arch. Oral Biol*. **2003**, 48, 423-9.

21. Vinolo, M.A.R.; Rodrigues, H.G.; Hatanaka, E.; Hebeda, C.B.; Farsky, S.H.P.; Curi, R. Short-chain fatty acids stimulate the migration of neutrophils to inflammatory sites. *Clin. Sci*. **2009**, 117, 331-8.

22. Wyss, C. Fatty acids synthesized by oral treponemes in chemically defined media. *FEMS Microbiol. Lett*. **2007**, 269, 70-6.

23. Yin, L.; Chung, W.O. Epigenetic regulation of human β-defensin 2 and CC chemokine ligand 20 expression in gingival epithelial cells in response to oral bacteria. *Mucosal Immunol*. **2011**, 4, 409-19.

24. Yoneda, S.; Kawarai, T.; Narisawa, N.; Tuna, E.B.; Sato, N.; Tsugane, T.; Saeki, Y.; Ochiai, K.; Senpuku, H. Effects of short-chain fatty acids on Actinomyces naeslundii biofilm formation. *Mol. Oral Microbiol*. **2013**, 28, 354-65.

25. Yoshida, Y.; Sato, M.; Nagano, K.; Hasegawa, Y.; Okamoto, T.; Yoshimura, F. Production of 4-hydroxybutyrate from succinate semialdehyde in butyrate biosynthesis in Porphyromonas gingivalis. *Biochim. Biophys. Acta*. **2015**, 1850, 2582-91.
