# Supplementary material for: Effects of Short-Chain Fatty Acids on Human Oral Epithelial Cells and the Potential Impact on Periodontal Disease: A Systematic Review of In Vitro Studies
Source: Int J Mol Sci. 2020 Jul 11;21(14):4895. doi: 10.3390/ijms21144895 (PMC7402343; doi:10.3390/ijms21144895)
Supplement: Supplementary file 1 [file ijms-21-04895-s001.zip › Table S2.docx]

**Table S2 –** Data search strategy.

| **Database** | **Search query**  2020, June 10^th^ |
| --- | --- |
| **PubMed** | (("epithelial cells"[MeSH Terms] OR "epithelial cells"[Title/Abstract] OR "gingival epithelial cell"[Title/Abstract] OR "gingival epithelial cells"[Title/Abstract] OR "oral epithelial cell"[Title/Abstract] OR "oral epithelial cells"[Title/Abstract] OR keratinocyte*[Title/Abstract] OR squamous cell[MeSH Terms] OR squamous cell[Title/Abstract] OR "junctional epithelium"[Title/Abstract] OR "junctional epithelia"[Title/Abstract] OR epithelial barrier[Title/Abstract] OR periodont*[Title/Abstract] OR oral[Title/Abstract] OR periodontium[MeSH Terms] OR gingiva[MeSH Terms] OR mouth[MeSH Terms] OR mouth[Title/Abstract] OR gingiva*[Title/Abstract]) AND ("volatile fatty acids" [MeSH Terms] OR "volatile fatty acids" [Title/Abstract] OR "short chain fatty acid"[Title/Abstract] OR "short chain fatty acids"[Title/Abstract] OR "short chain carboxylic acid"[Title/Abstract] OR "short chain carboxylic acids"[Title/Abstract] OR butyrates[MeSH Terms] OR butyrate*[Title/Abstract] OR "butyric acid"[Title/Abstract] OR "butyric acids"[Title/Abstract] OR Acetates[MeSH Terms] OR acetate*[Title/Abstract] OR "acetic acid"[Title/Abstract] OR "acetic acids"[Title/Abstract] OR propionates[MeSH Terms] OR propionate*[Title/Abstract] OR "propionic acid"[Title/Abstract] OR "propionic acids"[Title/Abstract] OR caproates[MeSH Terms] OR caproate*[Title/Abstract] OR "caproic acid"[Title/Abstract] OR "caproic acids"[Title/Abstract] OR valerates[MeSH Terms] OR valerate*[Title/Abstract] OR "valeric acid"[Title/Abstract] OR "valeric acids"[Title/Abstract] OR "pentanoic acid"[Title/Abstract] OR "pentanoic acids"[Title/Abstract])) AND ("cell adhesion molecule" [MeSH Terms] OR "cell adhesion molecule" [Title/Abstract] OR "intercellular adhesion molecule" [Title/Abstract] OR "adhesion molecule"[Title/Abstract] OR "intercellular junction"[Title/Abstract] OR "intercellular junctions"[Title/Abstract] OR "periodontal diseases" [MeSH Terms] OR "periodontal diseases" [Title/Abstract] OR periodontitis[Title/Abstract]) |
| **LILACS** | tw:("epithelial cell" OR "epithelial cells" OR "gingival epithelial cell" OR "gingival epithelial cells" OR "oral epithelial cell" OR "oral epithelial cells" OR "keratinocyte" OR "keratinocytes" OR "squamous cell" OR "squamous cells" OR "junctional epithelium" OR "junctional epithelia" OR "epithelial barrier" OR "periodontal" OR "oral" OR "periodontium" OR "gingiva" OR "gingival" OR "mouth" or "células epiteliales" OR "células epiteliais" OR "queratinocitos" OR "queratinócitos" OR "inserción epitelial" OR "inserção epitelial" OR "periodoncio" OR "periodonto" OR "encía" OR "gengiva" OR "gengival" OR "boca")) AND (tw:("volatile fatty acid" OR "volatile fatty acids" OR "short chain fatty acid" OR "short chain fatty acids" OR "short chain carboxylic acid" OR "short chain carboxylic acids" OR "butyrate" OR "butyrates" OR "butyric acid" OR "butyric acids" OR "acetate" OR "acetates" OR "acetic acid" OR "acetic acids" OR "propionate" OR "propionates" OR "propionic acid" OR "propionic acids" OR "caproate" OR "caproates" OR "caproic acid" OR "caproic acids" OR "valerate" OR "valerates" OR "valeric acid" OR "valeric acids" OR "pentanoic acid" OR "pentanoic acids" or "ácidos grasos volátiles" OR "ácidos graxos voláteis" OR "butirato" OR "butiratos" OR "ácido butírico" OR "acetato" OR "acetatos" OR "ácido acético" OR "propionato" OR "propionatos" OR "caproato" OR "caproatos" OR "valerato" OR "valeratos" OR "ácidos pentanoicos")) AND (tw:("cell adhesion molecule" OR "cell adhesion molecules" OR "intercellular adhesion molecule" OR "intercellular adhesion molecules" OR "adhesion molecule" OR "adhesion molecules" OR "intercellular junction" OR "intercellular junctions" OR "periodontal disease" OR "periodontal diseases" OR "periodontitis" or "moléculas de adhesión celular" OR "moléculas de adesão celular" OR "uniones intercelulares" OR "junções intercelulares" OR "enfermedades periodontales" OR "doenças periodontais" OR "periodontite") |
| **Web of Science** | #1 = ("epithelial cell" OR "epithelial cells" OR "gingival epithelial cell" OR "gingival epithelial cells" OR "oral epithelial cell" OR "oral epithelial cells" OR keratinocyte* OR "squamous cell" OR "squamous cells" OR "junctional epithelium" OR "junctional epithelia" OR "epithelial barrier" OR periodont* OR oral OR gingiva* OR mouth)  #2 = ("volatile fatty acid" OR "volatile fatty acids" OR "short chain fatty acid" OR "short chain fatty acids" OR "short chain carboxylic acid" OR "short chain carboxylic acids" OR butyrate* OR "butyric acid" OR "butyric acids" OR acetate* OR "acetic acid" OR "acetic acids" OR propionate* OR "propionic acid" OR "propionic acids" OR caproate* OR "caproic acid" OR "caproic acids" OR valerate* OR "valeric acid" OR "valeric acids" OR "pentanoic acid" OR "pentanoic acids")  #3 = ("cell adhesion molecule" OR "cell adhesion molecules" OR "intercellular adhesion molecule" OR "intercellular adhesion molecules" OR "adhesion molecule" OR "intercellular junction" OR "intercellular junctions" OR "periodontal disease" OR "periodontal diseases" OR periodontitis)  #4 = #3 AND #2 AND #1 |
| **EMBASE** | ('epithelial cell'/exp OR 'epithelial cells'/exp OR 'gingival epithelial cell'/exp OR 'gingival epithelial cells' OR 'oral epithelial cell' OR 'oral epithelial cells' OR keratinocyte* OR 'squamous cell'/exp OR 'squamous cells' OR 'junctional epithelium' OR 'junctional epithelia' OR 'epithelial barrier'/exp OR periodont* OR oral OR gingiva* OR 'mouth'/exp) AND ('volatile fatty acid'/exp OR 'volatile fatty acids' OR 'short chain fatty acid'/exp OR 'short chain fatty acids' OR 'short chain carboxylic acid' OR 'short chain carboxylic acids' OR butyrate* OR 'butyric acid'/exp OR 'butyric acids'/exp OR acetate* OR 'acetic acid'/exp OR 'acetic acids'/exp OR propionate* OR 'propionic acid'/exp OR 'propionic acids'/exp OR caproate* OR 'caproic acid'/exp OR 'caproic acids' OR valerate* OR 'valeric acid'/exp OR 'valeric acids' OR 'pentanoic acid'/exp OR 'pentanoic acids'/exp) AND ('cell adhesion molecule'/exp OR 'cell adhesion molecules'/exp OR 'intercellular adhesion molecule'/exp OR 'intercellular adhesion molecules' OR 'adhesion molecule'/exp OR 'intercellular junction'/exp OR 'intercellular junctions'/exp OR 'periodontal disease'/exp OR 'periodontal diseases'/exp OR 'periodontitis'/exp) |
| **SCOPUS** | ( TITLE-ABS-KEY ( "epithelial cell" OR "epithelial cells" OR "gingival epithelial cell" OR "gingival epithelial cells" OR "oral epithelial cell" OR "oral epithelial cells" OR keratinocyte* OR "squamous cell" OR "squamous cells" OR "junctional epithelium" OR "junctional epithelia" OR "epithelial barrier" OR periodont* OR oral OR gingiva* OR mouth ) ) AND ( TITLE-ABS-KEY ( "volatile fatty acid" OR "volatile fatty acids" OR "short chain fatty acid" OR "short chain fatty acids" OR "short chain carboxylic acid" OR "short chain carboxylic acids" OR butyrate* OR "butyric acid" OR "butyric acids" OR acetate* OR "acetic acid" OR "acetic acids" OR propionate* OR "propionic acid" OR "propionic acids" OR caproate* OR "caproic acid" OR "caproic acids" OR valerate* OR "valeric acid" OR "valeric acids" OR "pentanoic acid" OR "pentanoic acids" ) ) AND ( TITLE-ABS-KEY ( "cell adhesion molecule" OR "cell adhesion molecules" OR "intercellular adhesion molecule" OR "intercellular adhesion molecules" OR "adhesion molecule" OR "intercellular junction" OR "intercellular junctions" OR "periodontal disease" OR "periodontal diseases" OR periodontitis ) ) |
| **Grey Literature** | |
| **Google Scholar** | ("epithelial cells" OR "gingival epithelial cells" OR "oral epithelial cells" OR keratinocytes OR "squamous cells" OR "junctional epithelium" OR "epithelial barrier" OR periodontal OR oral OR periodontium OR gingiva OR mouth) AND ("volatile fatty acids" OR "short chain fatty acids" OR "short chain carboxylic acids" OR "Butyric acid" OR "acetates OR "acetic acid" OR propionates OR "propionic acid" OR caproates OR "caproic acid" OR "valeric acid" OR "pentanoic acids") AND ("cell adhesion molecules" OR "intercellular adhesion molecules" OR "adhesion molecule" OR "intercellular junctions" OR "periodontal disease" OR "periodontal diseases" OR periodontitis) |
| **Open Grey** | ("epithelial cells" OR "gingival epithelial cells" OR "oral epithelial cells" OR keratinocytes OR "squamous cells" OR "junctional epithelium" OR "epithelial barrier" OR periodontal OR oral OR periodontium OR gingiva OR mouth) AND ("volatile fatty acids" OR "short chain fatty acids" OR "short chain carboxylic acids" OR "Butyric acid" OR "acetates OR "acetic acid" OR propionates OR "propionic acid" OR caproates OR "caproic acid" OR "valeric acid" OR "pentanoic acids") AND ("cell adhesion molecules" OR "intercellular adhesion molecules" OR "adhesion molecule" OR "intercellular junctions" OR "periodontal disease" OR "periodontal diseases" OR periodontitis) |
| **ProQuest** | noft("epithelial cells" OR "gingival epithelial cells" OR "oral epithelial cells" OR "keratinocytes" OR "squamous cells" OR "junctional epithelium" OR "epithelial barrier" OR "periodontal" OR "oral" OR "periodontium" OR "gingiva" OR "mouth") AND noft("volatile fatty acids" OR "short chain fatty acids" OR "short chain carboxylic acids" OR "butyric acid" OR "acetates" OR "acetic acid" OR "propionates" OR "propionic acid" OR "caproates" OR "caproic acid" OR "valeric acid" OR "pentanoic acids") AND noft("cell adhesion molecules" OR "intercellular adhesion molecules" OR "adhesion molecule" OR "intercellular junctions" OR "periodontal diseases" OR "periodontitis") |
